# Adherence and Population Pharmacokinetic Properties of Amodiaquine When Used for Seasonal Malaria Chemoprevention in African Children

**DOI:** 10.1002/cpt.1707

**Published:** 2019-12-31

**Authors:** Junjie Ding, Matthew E. Coldiron, Bachir Assao, Ousmane Guindo, Daniel Blessborn, Markus Winterberg, Rebecca F. Grais, Alena Koscalova, Celine Langendorf, Joel Tarning

**Affiliations:** ^1^ Centre for Tropical Medicine and Global Health Nuffield Department of Clinical Medicine University of Oxford Oxford UK; ^2^ The WorldWide Antimalarial Resistance Network Oxford UK; ^3^ Children's Hospital of Fudan University Shanghai China; ^4^ Epicentre Paris France; ^5^ Epicentre Maradi Niger; ^6^ Mahidol‐Oxford Tropical Medicine Research Unit Faculty of Tropical Medicine Mahidol University Bangkok Thailand; ^7^ Médecins Sans Frontières Geneva Switzerland

## Abstract

Poor adherence to seasonal malaria chemoprevention (SMC) might affect the protective effectiveness of SMC. Here, we evaluated the population pharmacokinetic properties of amodiaquine and its active metabolite, desethylamodiaquine, in children receiving SMC under directly observed ideal conditions (*n* = 136), and the adherence of SMC at an implementation phase in children participating in a case‐control study to evaluate SMC effectiveness (*n* = 869). Amodiaquine and desethylamodiaquine concentration‐time profiles were described simultaneously by two‐compartment and three‐compartment disposition models, respectively. The developed methodology to evaluate adherence showed a sensitivity of 65–71% when the first dose of SMC was directly observed and 71–73% when no doses were observed in a routine programmatic setting. Adherence simulations and measured desethylamodiaquine concentrations in the case‐control children showed complete adherence (all doses taken) in < 20% of children. This result suggests that more efforts are needed urgently to improve the adherence to SMC among children in this area.

**Study Highlights**

**WHAT IS THE CURRENT KNOWLEDGE ON THIS TOPIC?**

☑ Adherence may pose a problem during seasonal malaria chemoprevention (SMC). Patient self‐reporting may lead to an overestimation of adherence. Objective approaches to assess the adherence of SMC are needed.

**WHAT QUESTION DID THIS STUDY ADDRESS?**

☑ This study evaluated the pharmacokinetic (PK) properties of amodiaquine and its active metabolite desethylamodiaquine among children in Niger, and the level of adherence in children receiving SMC.

**WHAT DOES THIS STUDY ADD TO OUR KNOWLEDGE?**

☑ Population PK properties of amodiaquine and desethylamodiaquine were characterized in young children (*n* = 136) receiving SMC under ideal, directly observed conditions in Niger. The developed PK model was used to develop a formal methodology to assess adherence in routine settings, based on modeling and simulation in combination with only one drug measurement per patient. This methodology was used to assess adherence in a group of children (*n* = 869) receiving SMC in the same area. Results showed complete adherence in < 20% of children.

**HOW MIGHT THIS CHANGE CLINICAL PHARMACOLOGY OR TRANSLATIONAL SCIENCE?**

☑ Adherence to SMC was poor in this setting and more efforts are needed to improve the adherence and the effectiveness of SMC in these children.

Malaria transmission is highly seasonal in the Sahel, where it represents a major health problem, particularly for children under 5 years of age. Seasonal malaria chemoprevention (SMC) has been recommended by the World Health Organization (WHO) as a preventive strategy since 2013.^1^ It consists of the monthly administration of antimalarial medications (sulfadoxine‐pyrimethamine (SP) + amodiaquine (AQ)), during the 4‐month peak transmission period.^1^ A single dose of SP is given together with the first dose of AQ followed by daily dosing of AQ for 2 subsequent days. Distribution strategies vary, but commonly the first day's treatments are given by a health worker and the two doses of AQ are given at home by a caregiver.

The goal of SMC is to prevent malaria by maintaining therapeutic concentrations of antimalarial drugs during the high‐risk period.^2^ AQ has a relatively short terminal elimination half‐life of 3.3–28 hours and is quickly metabolized into desethylamodiaquine (DEAQ), mediated by cytochrome P450 2C8 (CYP2C8).^3^, ^4^ DEAQ is an active metabolite with a relatively long terminal elimination half‐life (4–9 days),^5^, ^6^, ^7^, ^8^, ^9^ comparable to that of SP (6–7 and 3–5 days for sulfadoxine and pyrimethamine, respectively) reported in pediatric populations.^10^, ^11^, ^12^, ^13^ A meta‐analysis based on six clinical trials in West Africa estimated that ~ 75% of uncomplicated and severe malaria were avoided due to SMC.^14^ SMC has also been shown to reduce the prevalence of asymptomatic parasitemia at the end of the transmission season.^15^

In conjunction with the Ministry of Public Health of Niger, Médecins Sans Frontières began implementing SMC in the Magaria District of Niger in 2013. Despite good program coverage and considerable efforts of community mobilization and sensitization around the importance of good adherence to SMC, the incidence of malaria in health centers in the area remained high. Thus, questions were raised regarding the protective effectiveness of SMC in this area, and a case‐control study was implemented during the 2016 SMC season. Poor adherence to the full course of SMC was suggested as a plausible hypothesis to the observed high incidence of malaria.

According to taxonomy on medication adherence, different levels of adherence to SMC could occur at the initiation of treatment (child does not initiate treatment) and/or in the implementation phase of the treatment (e.g., delays or omission of doses).^16^ However, in the setting of SMC, the first dose is commonly observed and nonadherence would mostly concern the implementation phase, in which the child/caretaker delays or omits doses in favor of another child being ill or the expectation of an acute malaria episode at a later time point.

To the best of our knowledge, information on the population pharmacokinetic (PK) properties of AQ and DEAQ are limited in young children,^5^, ^6^, ^7^, ^8^ and has not been described when coadministered with SP in the setting of SMC. Moreover, information on adherence to SMC drugs is sparse and commonly based on self‐report. Adherence to curative antimalarial treatments has been reported in a number of clinical trials, and a systematic review of 55 published clinical trials showed that adherence to artemisinin‐based combination therapies ranged between 1.5% and 100%, using indirect adherence methods (self‐report, questionnaire, pill count, or a combination of these).^17^ However, patient self‐report may lead to an overestimation of adherence.^18^ Direct adherence assessment approaches of measuring blood/plasma concentrations of drug and/or its metabolites are thought to be more objective. In theory, drug concentration‐time profiles are different in fully adherent and nonadherent individuals, resulting in a different distribution of concentration values at any given sampling time.^19^, ^20^

The aim of this study was to evaluate adherence in young children receiving SP‐AQ in an SMC setting in Niger. In order to assess adherence, we designed a study to characterize the population PK properties of both AQ and DEAQ in the SMC target population (“PK cohort”). The developed model was used to derive concentration thresholds for different degrees of nonadherence and then used in combination with a single drug measurement in a large case‐control study to assess adherence to SMC objectively in this region.

## Results

### Population PK of AQ and DEAQ in the PK cohort

**Figure 1:**
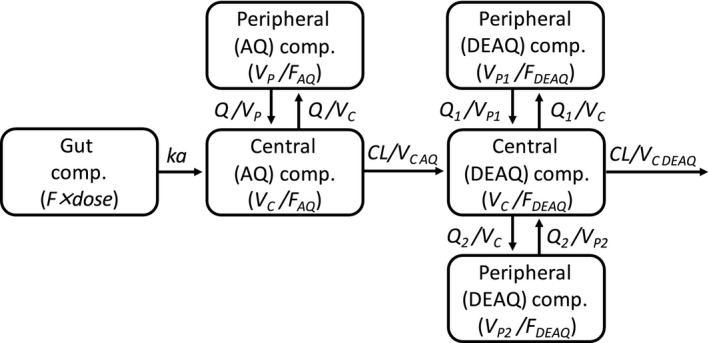
Graphical overview of the structural pharmacokinetic model for amodiaquine and desethylamodiaquine. AQ, amodiaquine; CL, elimination clearance; DEAQ, desethylamodiaquine; *F*, the relative oral bioavailability; *k*_a_, first‐order absorption rate constant; *Q*, intercompartmental clearance; *V*_C_, central volume of distribution; *V*_P_, peripheral volume.

**Figure 2:**
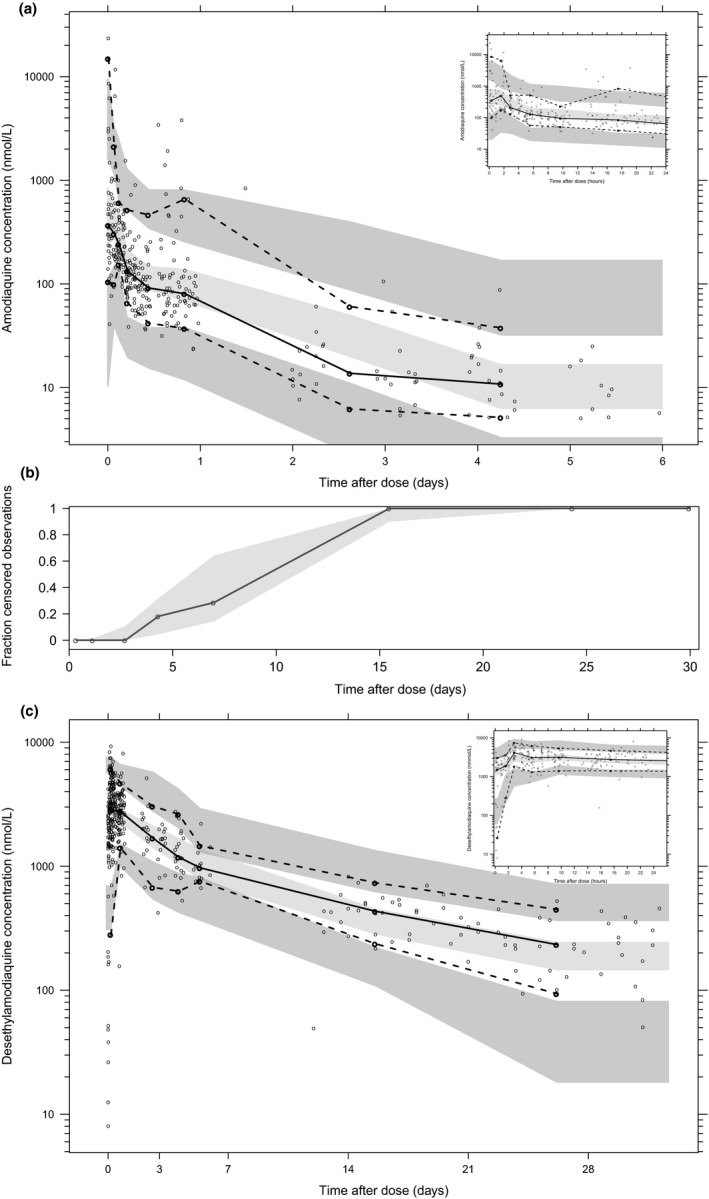
Visual predictive check of the final population pharmacokinetic model for amodiaquine (**a**, **b**) and desethylamodiaquine (**c**) based on 2,000 stochastic simulations. **a** and **c**: Open circles represent the observations, and lines represent the 5th, 50th, and 95th percentiles of the observed data. The shaded areas represent the 95% confidence intervals around the simulated 5th, 50th, and 95th percentiles. Inserts show the predictive performance during the first 24 hours of treatment. **b**: Open circles represent the observed fraction of censored data, and the shaded area represent the 95% confidence interval of the simulated fraction of censored data.

AQ and DEAQ concentration‐time profiles were best described by two and three disposition compartments, respectively, with first‐order absorption and first‐order elimination (**Figure** 1). A categorical visual predictive check for censored data (**Figure** 2) showed good agreement between predicted and observed data below the lower limit of quantification (LLOQ), when omitting LLOQ data from the analysis (M1 method). Using a more complex approach to handle LLOQ data, such as the M3 method (likelihood estimation) or the M6 method (imputations), was not necessary and, therefore, not evaluated further. Furthermore, none of the DEAQ concentrations was measured to be below the LLOQ, further supporting a parsimonious method of omitting LLOQ data. Thus, all LLOQ samples were omitted when evaluating the population PK properties of AQ and DEAQ.

Predicted body weight, implemented as a fixed allometric function on all clearance and volume of distribution parameters, showed a substantial improvement in model fit (∆ objective function volume (OFV) = −14.8). An age‐dependent enzyme maturation effect on AQ clearance showed a significant improvement in model fit (∆OFV = −14.7; *P* < 0.001), with further improvement when implementing this also on DEAQ (∆OFV = −7.11; *P* < 0.01). Half of full enzyme maturation (age_50_) was reached after 4.7 and 2.4 months for AQ and DEAQ, respectively.

Predicted weight‐for‐age Z‐score (WAZ) was significantly associated with relative bioavailability in a linear manner, with a 38.3% decrease in relative bioavailability per 1 unit WAZ decrease (∆OFV = −7.5; *P* < 0.01). However, this parameter was estimated with poor precision (i.e., high relative standard error of 56%), suggesting the model to be overparameterized when predicted WAZ was included in the model. WAZ was, therefore, not included in the final model.

Dosage (mg/kg) and sex did not have a significant impact on the PK properties of AQ or DEAQ. Thus, after receiving identical dosages (mg/kg), these covariates resulted in a relatively higher exposure to AQ and DEAQ in infants and toddlers due to the age‐dependent maturation effects (**Figure** **S1** **a**), and a relatively lower exposure to AQ and DEAQ in children with a low bodyweight due to allometric scaling of body weight (**Figure** **S1** **b**).

The final parameter estimates of AQ and DEAQ showed good precision with relatively small standard errors (**Table** 1), confirming the stability of the model, and providing confidence when using the developed population PK model to simulate different adherence scenarios. The final parameter estimates described the expected absorption, distribution, and elimination processes, as well as the associated unexplained variability of both AQ and DEAQ in children under 5 years. Secondary parameters describing the exposure to AQ and DEAQ (i.e., peak concentration, half‐life, and area under the curve (AUC)), were derived from Empirical Bayes Estimates (**Table** 1). Goodness‐of‐fit diagnostic plots (**Figure** **S2**) and visual predictive checks (**Figure** 2) demonstrated good description of observed data and adequate predictive performance of the final model. The complete final population PK model code can be found in the **Supplementary Material**.

**Table 1:** Final population PK parameter estimates of AQ and DEAQ in children aged 3‐59 months

Parameter	NONMEM population estimates (%RSE)	SIR median (95% CI)	CV for IIV (%RSE)	SIR median (95% CI)	Shrinkage (%)
AQ					
*F*_AQ_ (%)	100 *fix*	–	37.5 (7.1)	37.2 (32.1–42.5)	8.0
*k*_a_ (1/hour)	2.85 (42.8)	3.09 (1.71–5.70)	173 (26.0)	179 (131–237)	42.8
CL/*F*_AQ_ (L/hour)	101 (12.3)	100 (85–120)	22.2 (35.2)	23.1 (7.61–33.0)	47.4
*V*_C_/*F*_AQ_ (L)	314 (22.1)	309 (214–445)	80.4 (15.9)	80.5 (56.0–104)	42.8
*Q*/*F*_AQ_ (L/hour)	119 (27.3)	115 (84.7–161)	–	–	–
*V*_P_/*F*_AQ_ (L)	1,820 (15.8)	1,772 (1,460–2,220)	–	–	–
σ_AQ_	0.829 (5.9)	0.831 (0.742–0.920)	–		–
DEAQ					
CL/*F*_DEAQ_ (L/hour)	2.33 (7.8)	2.32 (2.08–2.57)	15.2 (61.5)	16.1 (4.05–26.9)	54.0
*V*_C_/*F*_DEAQ_ (L)	49.1 (17.6)	49.3 (39.2–61.5)	–	–	–
*Q*_1_/*F*_DEAQ_ (L/hour)	2.31 (29.9)	2.23 (1.54–3.42)	–	–	–
*V*_P1_/*F*_DEAQ_ (L)	363 (16.9)	355 (293–462)	68.3 (30.9)	69.1 (55.0–84.7)	46.2
*Q*_2_/*F*_DEAQ_ (L/hour)	4.34 (47.2)	4.45 (2.51–6.86)	–	–	–
*V*_P2_/*F*_DEAQ_ (L)	98.1 (42.9)	100 (46.1–153)	–	–	–
σ_DEAQ_	0.204 (8.8)	0.201 (0.181–0.233)	–	–	–
Covariate relationships					
Age_50_ on CL/*F*_AQ_ (months)	4.66 (40.1)	4.60 (1.92–8.08)	–	–	–
Age_50_ on CL/*F*_DEAQ_ (months)	2.42 (49.6)	2.45 (0.838–4.47)	–	–	–
Secondary parameters					
*C*_max AQ_ (nmol/L)	835 (73.8–6,916)				
*t*_1/2 AQ_ (hour)	27.1 (23.5–91.2)				
AUC_0‐∞ AQ_ (hour × μmol/L)	14.76 (9.30–60.6)				
*C*_max DEAQ_ (nmol/L)	3,272 (465–7,860)				
*t*_1/2 DEAQ_ (day)	11.0 (3.06–30.8)				
AUC_0‐∞ DEAQ_ (hour × μmol/L)	576 (399–1,743)				

σ, additive residual error on log scale; Age_50_, age associated with 50% of clearance maturity; AQ, amodiaquine; AUC_0‐∞_, area under the concentration‐time curve from time zero to infinity; CI, confidence interval; CL/F, elimination clearance; *C*_max_, maximum concentration; DEAQ, desethylamodiaquine; *F*, relative bioavailability; IIV, interindividual variability; *k*_a_, absorption rate constant; PK, pharmacokinetic; *Q*/*F*, intercompartmental clearance; RSE, relative standard error; SIR, sampling importance resampling; *t*_1/2_, terminal elimination half‐life; *V*_C_/*F*, central volume of distribution; *V*_P_/*F*, peripheral volume of distribution.

Secondary‐parameter estimates were calculated from the Empirical Bayes *post hoc* estimates and presented as median (range).

Population estimates in the table are given for a “typical” child with bodyweight of 10 kg and full maturation of metabolizing enzymes.

Coefficients of variation for IIV were calculated as 100 × (e^variance^)^1/2^. %RSEs were calculated as 100 × (SD/mean). Age was implemented using a maturation model on CL $\left({\text{CL}}_{i}={\text{CL}}_{\text{TV}}\times {\left(\frac{\text{Bodyweight}}{10}\right)}^{0.75}\times \left(\frac{\text{Age}}{{\text{Age}}_{50}+\text{Age}}\right)\right)$, where CL_i_ is the individually predicted clearance and CL_TV_ is the typical clearance value of the population. The uncertainties were derived from SIR with options of 2,000 samples and 1,000 resamples.

### Predictive performance of the adherence method

**Figure 3:**
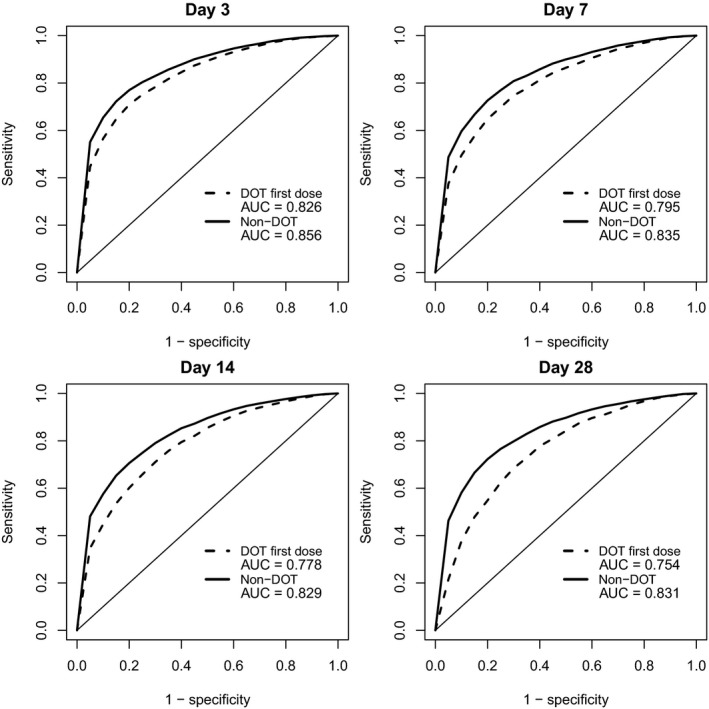
The receiver operating characteristic curve of desethylamodiaquine concentration at different time for assessment of adherence. AUC, area under the curve; DOT, direct observed therapy.

The population PK‐based percentile approach for assessing adherence demonstrated a receiver operating characteristic curve above 0.75 and 0.80 for first‐dose directly observed therapy (DOT) and non‐DOT regimens, respectively, suggesting a fair discriminating capacity (**Figure** 3). The optimal cutoff value, according to Youden's index,^21^ was the 20th, 25th, and 30th percentiles for DEAQ concentrations measured at days 3–7, days 8–21, and days 22–30, respectively, for the DOT regimen. The sensitivity was 71%, 65%, 66%, and 68%, along with a specificity of 80%, 80%, 75%, and 70%, for DEAQ concentrations measured at days 3, 7, 14, and 28, respectively. For the non‐DOT regimen, the optimal percentile cutoff value was 20% and 25% for DEAQ concentrations measured at days 3–21 and days 22–30, respectively. The sensitivity was 77%, 73%, 71%, and 72%, along with a specificity of 80%, 80%, 80%, and 75%, for DEAQ concentrations measured at days 3, 7, 14, and 28, respectively. These optimal percentile cutoff values were applied to assess adherence in case‐control study participants. For a more conservative approach, we also assessed the adherence when assuming a cutoff value at the fifth percentile (specificity was 95%). The sensitivity was 22–45% and 35–55% for DOT and non‐DOT regimens, respectively, when using the fifth percentile cutoff value. A sensitivity analysis showed a minor impact on the percentile cutoff values associated with nonadherence scenarios due to errors in dose timing (e.g., < 23% relative difference and ~ 10 nmol/L absolute difference in the fifth percentile cutoff value at day 28, when comparing full adherence with an extreme scenario of all three doses administrated on day 1).

### Adherence to SMC in children enrolled in the case‐control study

In the case‐control study, a total of 1,146 children aged 4–57 months had samples analyzed for AQ and DEAQ drug concentrations, including 287 malaria cases and 859 matched community controls. Of these children, 869 of 1,146 (75.8%) self‐reported as having complete adherence (193/287 (67.2%) of cases and 676/859 (78.7%) of community controls). Among children with reported correct adherence in the control group, 327 of 676 (48.4%) resided in a DOT zone and 349 of 676 (51.6%) resided in a non‐DOT zone. A total of 96 of 327 (29.4%) and 145 of 349 (41.5%) of children in DOT and non‐DOT zones, respectively, had undetectable DEAQ concentrations at enrollment (within 1 month after the most recent SMC distribution). Similar trends were seen in the malaria cases group. The results from the adherence evaluation, using the percentile method, demonstrated that > 70% of children demonstrated poor adherence during each of the four rounds of SMC, irrespectively of what percentile cutoff value that was used (**Table** 2). Sex and age were comparable between children in the DOT and non‐DOT zones in the case‐control study and between children in the case‐control study and in the PK cohort.

**Table 2:** Adherence assessment by the percentile methods

Round of SMC	Estimated complete adherence	
	Fifth percentile cutoff	Optimal percentile cutoff
Community controls		
First‐dose DOT		
Round 1 (*n* = 70)	19 (27.1%)	10 (14.3%)
Round 2 (*n* = 72)	9 (12.5%)	5 (6.9%)
Round 3 (*n* = 99)	12 (12.1%)	8 (8.1%)
Round 4 (*n* = 86)	15 (17.4%)	4 (4.7%)
Total (*n* = 327)	55 (16.9%)	27 (8.3%)
First‐dose non‐DOT		
Round 1 (*n* = 63)	14 (22.2%)	3 (4.8%)
Round 2 (*n* = 101)	20 (19.8%)	15 (14.9%)
Round 3 (*n* = 89)	15 (16.9%)	9 (10.1%)
Round 4 (*n* = 96)	12 (12.5%)	2 (2.1%)
Total (*n* = 349)	61 (17.5%)	29 (8.3%)
Malaria cases		
First‐dose DOT		
Round 1 (*n* = 15)	2 (13.3%)	2 (13.3%)
Round 2 (*n* = 19)	2 (10.5%)	1 (5.3%)
Round 3 (*n* = 24)	3 (12.5%)	2 (8.3%)
Round 4 (*n* = 26)	1 (3.8%)	1 (3.8%)
Total (*n* = 84)	8 (9.5%)	6 (7.1%)
First‐dose non‐DOT		
Round 1 (*n* = 18)	1 (5.6%)	1 (5.6%)
Round 2 (*n* = 33)	2 (6.1%)	1 (3.0%)
Round 3 (*n* = 28)	1 (3.6%)	0 (0%)
Round 4 (*n* = 30)	1 (3.3%)	1 (3.3%)
Total (*n* = 109)	5 (4.6%)	3 (2.8%)

Data are reported as *n* (%). The SMC was distributed monthly from July to October. Each distribution was named “round,” resulting in four rounds of SMC representing the first to fourth monthly distribution of SMC. Results were derived based on the assumption that children took the drug at scheduled times.

DOT, directly observed therapy; SMC, seasonal malaria chemoprevention.

## Discussion

Monthly SMC with SP‐AQ is now recommended by the WHO for children aged 3–59 months living in regions with high seasonal malaria transmission.^1^ To the best of our knowledge, this is the first description of the population PK of AQ when it is used in the setting of SMC, and the first assessment of adherence to SMC treatment using this method. The population PK model developed for AQ and DEAQ in children aged 3–59 months was robust and showed good predictive performance. In the current study, we developed a parent‐metabolite model to fit the concentration‐time profiles of AQ and DEAQ simultaneously. The resulting multiphasic disposition model resulted in an average (range) terminal elimination half‐life of AQ and DEAQ of 27.1 (23.5–91.5) hours and 11.0 (3.1–30.8) days, respectively. This is similar to previously published results of 3.3–28.4 hours for AQ and 5–12 days for DEAQ.^5^, ^6^, ^7^, ^8^, ^9^, ^22^, ^23^ This slow elimination of DEAQ causes an accumulation of drug concentrations with each subsequent round of SMC, a drug property that can be used successfully for adherence monitoring. We found that bodyweight and age had a significant impact on the PK properties. This impact has been described in a previously published pooled pharmacometric analysis of AQ and DEAQ from six clinical trials, showing very similar maturation and bodyweight effects.^22^

Medication adherence may pose a problem during SMC, and poor adherence can lead to subtherapeutic drug concentrations, increasing the risk of malaria and development of drug resistance. To date, a number of direct and indirect methods have been developed to investigate medication adherence for either treatment of malaria or SMC. Self‐report, pill counts, or a combination of both are most feasible, relatively low cost, and shown to be the most useful indirect methods to estimate adherence in malaria therapy^17^, ^24^ and SMC.^25^, ^26^, ^27^ However, the response bias of self‐report (and to a lesser degree, recall bias) by patients may result in an overestimation of adherence.^18^ Moreover, the sensitivity and specificity of self‐reported adherence in patients with acute malaria have been rarely reported, with only one study showing very high sensitivity and specificity (> 96%), when considering smart blister packs as a reference standard,^28^ but is important to note that children receiving SMC are not acutely ill.

Self‐reported adherence to SMC has been reported as being high but the reliability regarding this is unclear. Diawara *et al.* 27 reported that the adherence to SMC was > 95% when self‐reported by caregivers. A randomized, placebo‐controlled trial of SMC in Ghana showed close to 100% self‐reported adherence to the 3‐day course of SMC in all research communities. However, some caregivers were found to have SMC tablets remaining that had not been administered.^26^ Although it was not in the setting of SMC, a study comparing different malaria preventive regimens in Ugandan children showed that adherence to a 3‐day course of dihydroartemisinin‐piperaquine was much higher when reported by the caregiver (~ 100%) compared with unbiased drug concentration measurements (52%).^25^ This last example parallels our results.

For a conservative approach, we assumed that all children in the case‐control cohort had missed all previous rounds of SMC, resulting in lower DEAQ cutoff concentrations associated with complete adherence. Nevertheless, we found that > 80% of healthy children, with reported full adherence by caregivers, had poor adherence when assigning the measured drug levels. This was true using either the conservative fifth percentile cutoff value or the optimal (20–30th) percentile cutoff value, both in DOT and non‐DOT zones. Furthermore, 29.4% of children in the DOT zone and 41.5% in the non‐DOT zone had undetectable levels of DEAQ at enrollment. When considering another dimension of nonadherence (i.e., errors in dosing time), the most extreme scenario of self‐administering all three AQ doses on the first day of SMC showed only a minor impact on the simulated cutoff values at day 28 (relative difference was < 25%) due to the very long half‐life of DEAQ. This suggests that the poor adherence reported here is unlikely to be due to errors in dose timing.

The reason for the poor adherence in this setting of SMC is unclear. We speculate that this might be a result of one or several different aspects: (i) children refusing to take medication; (ii) suboptimal health worker instructions; (iii) small children spitting out medication, even if it is dissolved; (iv) vomiting within 30 minutes of dosing; (v) caregiver saving medication for treatment of another family member with acute malaria later on; (vi) caregivers sharing/giving medication to older children who were not eligible for SMC; (vii) fathers not allowing the medicine to be taken; and (viii) fatigue of giving medication, particularly when the child is not sick. Obviously, more efforts are needed to improve the adherence and the effectiveness of SMC in this setting.

The population PK‐based approach, used in this current study, is a promising unbiased method for assessing adherence, but it requires costly and labor‐intensive drug measurements. However, this method could be combined with other indirect tools, such as self‐report, pill count, and/or questionnaires, to improve further on the assessment of adherence or to develop an algorithm for assessing adherence. If patient claims to be adherent, but has high risk or a strong suspicion of poor adherence, a drug concentration assessed by a population PK‐based approach could be used to clarify the adherence status. Such evaluations would be imperative to understand nonadherence and could provide the tools needed to improve adherence in these patients.

Our study has several key limitations. (i) The PK cohort was not randomly selected, but participants lived in the same area and overall setting as the patients included in the case‐control study, and had largely similar baseline characteristics to the community controls. (ii) Due to the lack of measured bodyweight, we used age to predict bodyweight with a derived relationship between age and bodyweight from a large population in Niger. (iii) Presence of the CYP2C8*2 polymorphism, associated with a reduced AQ clearance and increased exposure,^29^ seen in 11.5–18.3% of the African population,^4^, ^30^ were not tested in this study. (iv) Malnutrition can affect the PK characteristics, especially absorption,^10^, ^31^, ^32^, ^33^ but anthropometric indicators such as mid‐upper arm circumference or z‐scores were not collected in the PK cohort. Indeed, the estimated WAZ score for the study population, using the predicted average bodyweights and the WHO 2007 growth standard,^34^ resulted in a score ranging from −2.13 to −0.86, suggesting a fair degree of malnutrition in this study population. However, predicted WAZ score was not retained as a covariate in the final model due to the poor precision when estimating this parameter. Nevertheless, a population PK model including WAZ score as a covariate on the relative bioavailability resulted in slightly increased estimates of nonadherence in all age groups (data not shown). (v) We based our modeling on conservative assumptions, which influenced parameter estimates and, as such, the results presented here should be interpreted qualitatively as an indicator of the importance of PK modeling and drug measurements compared to self‐report when assessing adherence of SMC. (vi) Other factors relevant to medication adherence (e.g., additional caregiver and patient demographics) were not analyzed in this study.

## Conclusion

The population PK properties of AQ and DEAQ were described successfully in children in Niger. Adherence to SMC derived from population PK modeling and drug measurements in this study was much worse than self‐reported, suggesting more efforts are needed to improve the adherence to SMC among children in this area.

## Methods

### Study design and drug regimen

The first group of participants was recruited in a prospective case‐control study, in which the primary objective was to estimate the protective effectiveness of SMC. In this case‐control study, children aged 3–59 months who presented to a health structure with clinical malaria (fever plus a positive *Plasmodium* lactate dehydrogenase‐based rapid diagnostic test) were considered cases. Clinical malaria cases were confirmed by direct microscopy and included any *Plasmodium* species. Two different sets of controls were enrolled for each case patient (i.e., three community controls and three health center controls for each case). Full details on the enrollment of controls can be found in the **Supplementary Material**. The only exclusion criteria for controls were the presence of clinical or laboratory‐confirmed malaria. Participants in the case‐control study were eligible to receive each of the four monthly distributions of SMC between July and October, 2016. Children aged 3–11 months received 250/12.5 mg of SP in a single dose and 57.5 mg of AQ in 3 daily doses. Children aged 12–59 months received 500/25 mg of SP in a single dose and 153 mg of AQ in 3 daily doses. The first dose of AQ was given at the same time as the dose of SP. In the case‐control study area, SMC distributions were carried out using two distinct strategies in two distinct zones: (i) the dose of SP and first dose of AQ were directly observed by a health worker (DOT zone) with the following two doses of AQ given by caregivers at home and (ii) the complete SMC blister pack was given to the child's caretaker with instructions on how to administer the medications at home (non‐DOT zone). The case‐control study was stratified by the two intervention zones. If the participant reported receiving the most recent distribution of SMC, caretakers were asked to self‐report adherence to the course at home. A total of 577 cases, 1,700 community controls, and 1,233 health‐center controls were enrolled in the case‐control study. For adherence evaluation, one‐half of the cases (*n* = 287) were randomly selected to have samples analyzed for drug levels, along with their age‐matched community‐controls (*n* = 859), resulting in a total of 1,146 children. This study focused on medication adherence in the implementation phase of SMC.

In order to describe the PK properties of both AQ and DEAQ in children, we enrolled a convenience sample, based on proximity to Epicentre study center in Magaria. All children aged 3–59 months living in those households were eligible to be enrolled in the PK study (PK cohort). Children enrolled in the PK cohort received SMC on November 14–16 or November 15–17, 2016, ~ 4.5 weeks after the fourth distribution of SMC, with all three doses of AQ delivered at the same time each day and administered with water under direct observations, at home, by a study nurse. In the PK cohort, the dosage regimen of AQ was identical to the case‐control population described above. Children who vomited a dose within 60 minutes after administration were retreated but excluded from the PK analysis. Children with fewer than two blood draws during the follow‐up period were also excluded from the PK analysis.

The study protocol was reviewed and approved by the National Consultative Ethics Committee of Niger and the Ethics Review Board of Médecins Sans Frontières. Parents or guardians were asked to provide written informed consent before enrollment of all participants. For this paper, ESPACOMP Medication Adherence Reporting Guideline (EMERGE) were followed.^35^

### Blood samples

For the PK cohort, each individual child had blood collected in three of six predefined sampling windows (0–8 hours after first dose, 0–6 hours after last dose, 6–12 hours after last dose, 12–24 hours after last dose, 4–7 days after first dose, and 14–35 days after first dose). In the case‐control study, one blood sample for each child was collected at the time of the enrollment. AQ and DEAQ drug concentrations were determined using a liquid‐chromatography tandem mass spectrometry‐based assay. Full details can be found in the **Supplementary Material**.

### Population PK analysis

A total of 136 children, aged 3–59 months, were included. Concentration‐time data for AQ and DEAQ (population PK) were evaluated simultaneously using nonlinear mixed‐effects modeling in the software NONMEM. Full details of the population PK analyses can be found in the **Supplementary Material**.

### Assessment the predictive performance of the adherence method

The adherence methodology was developed by simulating both adherent and nonadherent patient scenarios (**Table** 3), in order to conclude the optimal percentile cutoff value that was most discriminatory between adherent and nonadherent patients. The flowchart of the methodology for assessment of adherence is shown in **Figure** **S3**.

**Table 3:** Simulated adherence scenarios

	First‐dose DOT			First‐dose non‐DOT			Full adherence	Phase of nonadherence occurred
	First dose	Second dose	Third dose	First dose	Second dose	Third dose		
Scenario 1	√	√	√	√	√	√	Yes	–
Scenario 2	√	√	–	√	√	–	No	Implementation
Scenario 3	√	–	√	√	–	√	No	Implementation
Scenario 4	√	–	–	√	–	–	No	Implementation
Scenario 5				–	√	√	No	Implementation
Scenario 6				–	√	–	No	Implementation
Scenario 7				–	–	√	No	Implementation
Scenario 8				–	–	–	No	Initiation

The tick‐symbol represent patients taking the scheduled dose. A total of 2,000 individuals were simulated for each scenario, using final population pharmacokinetic model.

DOT, directly observed treatment.

Full adherence was defined as correctly taking all three doses of AQ in a previous round of SMC. A child missing at least one of the three doses of SMC was categorized as having poor adherence. AQ treatment in the latest round of SMC, results in 2^N^ possible combinations of dosing events, ranging from the full adherence (all doses taken) to complete nonadherence (all doses missed), where *N* is the number of nonobserved dose administrations. In the current study, *N* would be 2 for DOT zones and 3 for non‐DOT zones, generating 4 and 8 adherence scenarios, respectively (**Table** 3).

Because DEAQ has a long terminal elimination half‐live relative to AQ, adherence to the most recent round of SMC in participants of the case‐control study were assessed using measured DEAQ concentrations, and the developed population PK model. The developed population PK model for the PK cohort in this study was used to perform stochastic simulations (*n* = 2,000) for each adherence scenario in the DOT zone (four adherence scenarios, **Table** 3) and the non‐DOT zone (eight adherence scenarios, **Table** 3), using the deSolve package in R software (version 3.2.3). The simulations were based on a typical child aged 36 months old (median age of PK‐cohort) with an average bodyweight of 11.5 kg, receiving a daily dose of AQ (153 mg) for 3 days. The patient demographics used for the methodology development should have a negligible impact on the performance of the adherence assessment of the actual patients in the study. DEAQ cutoff concentrations at a given percentile value (e.g., 5%) were calculated at days 3, 7, 14, 21, and 28 after the first dose of AQ, simulating complete adherence (scenario 1).

Considering the simulated adherence as the gold standard, a two‐by‐two table was used to calculate sensitivity and specificity for different cutoff percentiles (5th to 95th, intervals of 5%). Full details of calculating sensitivity and specificity can be found in the **Supplementary Material**. Youden's index, defined as the sum of sensitivity and specificity minus 1, was used to assess the overall performance at different cutoff percentiles.^21^ A higher Youden's index suggests a better predictive performance. Additionally, a receiver operating characteristic curve was used to assess the overall predictive performance of the methodology by plotting [sensitivity] vs. [1 – specificity] for the different cutoff percentiles. In general, an AUC of 0.5 suggests no discriminating capacity, 0.7–0.8 is considered fair, 0.8–0.9 is considered excellent, and >0.9 is considered outstanding.^36^

### Adherence assessment among children enrolled in case‐control study

Because the case‐control study was conducted throughout the SMC season, and given that participants were eligible to receive one or more distributions prior to their enrollment in the study, there were too many simulation scenarios to assess. Therefore, we conservatively assumed that all case‐control participants had missed all previous rounds of SMC; the only round where self‐reported adherence was assessed was the most recent round prior to inclusion. If a child was reported to have vomited within 30 minutes of taking AQ at home, he was only considered to have full adherence if the caregiver sought a replacement dose at the nearest health center.

The developed population PK model was used to simulate 2,000 concentration‐time profiles of each child in the case‐control study (assuming full adherence) to calculate the cutoff concentration value at a given time (using the optimal cutoff percentile described above, as well as the conservative fifth percentile). The measured drug concentration for each child was then compared with the particular cutoff concentration for that patient, in order to conclude if the patient was likely to have adhered to the previous round of SMC dosing (**Figure** **S3**).

To investigate the impact of nonadherence due to dosing time errors, a simulation‐based sensitivity analysis was carried out. The most extreme scenario of self‐administering all three AQ doses on the first day of SMC was simulated and the fifth percentile cutoff value on day 28 was compared with the simulated cutoff value in individuals with complete adherence.

## Funding

Funding for the implementation of the PK cohort study, drug level measurements, and the case‐control study was provided by Médecins Sans Frontières. The work of the Department of Clinical Pharmacology, Mahidol‐Oxford Tropical Medicine Research Unit, is partly funded by the Wellcome Trust of Great Britain and the Bill & Melinda Gates Foundation. The funders had no part in the study design, implementation, and analysis of the result or the decision to publish this manuscript.

## Conflict of Interest

All other authors declared no competing interests for this work.

## Author Contributions

J.D., M.E.C., R.F.G., A.K., and J.T. wrote the manuscript. M.E.C. and J.T. designed the research. J.D., M.E.C., B.A., O.G., D.B., M.W., C.L., and J.T. performed the research. J.D and J.T. analyzed the data.

## Supporting information


**Figure S1.** The impact of age and bodyweight on the total exposure to desethylamodiaquine after a standard oral 3‐day dosing of amodiaquine (10 mg/kg/day).
**Figure S2.** Goodness‐of‐fit of the final population pharmacokinetic model describing amodiaquine (**a–c**) and desethylamodiaquine (**d–f**).
**Figure S3.** Flowchart of developed adherence methodology.

**Supplementary Material S1.** EMERGE‐guidelines‐Checklist.
