# Supplementary material for: Adherence and Population Pharmacokinetic Properties of Amodiaquine When Used for Seasonal Malaria Chemoprevention in African Children
Source: Clin Pharmacol Ther. 2019 Dec 31;107(5):1179–88. doi: 10.1002/cpt.1707 (PMC7232861; doi:10.1002/cpt.1707)
Supplement: Supplementary file 1 — Figure S1. The impact of age and bodyweight on the total exposure to desethylamodiaquine after a standard oral 3‐day dosing of amodiaquine (10 mg/kg/day). Figure S2. Goodness‐of‐fit of the final population pharmacokinetic model describing amodiaquine (a–c) and desethylamodiaquine (d–f). Figure S3. Flowchart of developed adherence methodology. [file CPT-107-1179-s001.docx]

***Adherence and population pharmacokinetic properties of amodiaquine when used for seasonal malaria chemoprevention in African children***

**SUPPLEMENTARY METHODS**

**Case-Control Study design**

Two different sets of controls were enrolled for each case patient (i.e. three community controls and three health center controls for each case). Three age-matched (±6 months) community controls were enrolled in the village of residence of the case patient on the same day of enrollment. Three age-matched (±6 months) health center-based controls were enrolled based on the same criteria as the community-based controls, but were recruited at the health center within a 72-hour window of the enrolment of the case patient.

**Blood samples**

For the PK cohort, scheduled in-home capillary blood draws continued from November 18 to December 23, 2016, with each child sampled at three separate time points over that period, approximately 34 days after the first dose of AQ. In order to capture the PK profile and minimize blood draws, each individual child had blood collected in 3 out of 6 pre-defined sampling windows (0-8 hours after first dose, 0-6 hours after last dose, 6-12 hours after last dose, 12-24 hours after last dose, 4-7 days after first dose, and 14-35 days after first dose).

In the case-control study, one blood sample for each child was collected at the time of the enrollment in order to assess the adherence of most recent round SMC.

In both the PK cohort and the case-control study, study nurses performed finger stick using self-retracting lancets. Capillary blood (0.5 mL) was collected into a microtube containing EDTA and transported to the study laboratory, where capillary blood was pipetted the same day onto filter paper (Whatman 31 ET CHR), dried and stored until shipment to the Department of Clinical Pharmacology, Mahidol Oxford Tropical Medicine Research Unit, Thailand for concentration measurements.

**Drug concentration quantification**

Drug concentrations were determined using an LC-MS/MS based assay, validated according to U.S. FDA guidelines. In brief, AQ and DEAQ were extracted from dried blood samples using a PHREE phospholipid removal plate (Phenomenex) and a Freedom EVO liquid handler system (Tecan). Stable isotope-labelled internal standards were used to compensate for potential recovery and matrix effects. The extracted drugs were separated using a Dionex Ultimate 3000 UHPLC (Thermo Fisher) equipped with a Zorbax SB-CN column (Agilent). An API 5000 triple-quadrupole mass spectrometer (ABSciex) and Analyst 1.7 software (ABSciex) were used for drug detection and quantification. The lower limit of quantification was 1.87 ng/mL for AQ and 2.95 ng/mL for DEAQ. Three replicates of quality control samples at low, middle, and high concentrations were included in each batch of clinical study samples to ensure precision and accuracy. Total precision (i.e. relative standard deviation) of all quality control samples was <5.47% during drug quantification of clinical samples.

**Data collection**

In the PK cohort, information about each participant’s age, gender, dose regimen (amount, date, time), and sampling date and time were collected in the field, and added to the capillary blood concentrations of both AQ and DEAQ after the assays were performed. The same information was collected from participants in the case-control study, but caregivers were simply asked whether the child had received the most recent distribution of SMC, and the date was inferred from the distribution schedule for the child’s village of origin. If a case-control study participant reported having received SMC, study staff asked a series of questions relating to how doses of AQ were taken at home.

**Population PK analysis**

One hundred and sixty-five children were enrolled in the PK cohort. Twelve children did not have complete follow-up (≤1 blood draw), and 17 children (10.9%) vomited within 60 minutes after dose. These children were excluded from the analysis. A total of 136 children, aged 3-59 months, were included in the analysis, of whom 78 (57.4%) were male. A total of 404 capillary blood samples were available for AQ and DEAQ concentration measurements. Samples collected ≥300 hours after dosing were excluded from the AQ analysis (i.e. 37 samples) due to the relatively short terminal elimination half-life of AQ (i.e. 3.3-28 hours^1–3^). 42 out of the remaining 367 (11.4%) AQ samples were below the LLOQ. None of the 404 samples for DEAQ measurement were measured to be below the LLOQ.

The population PK analysis was performed using nonlinear mixed-effects modelling in the software NONMEM (version 7.4, ICON Development Solutions, Ellicott City, MD, USA), compiled using gFortran (version 4.60). Perl-speaks-NONMEM (PsN; version 4.6.0) and R (version 3.2.0, http://www.r-project.org/) were used to evaluate the goodness of fit and output visualizations. The first-order conditional estimation method including η-ε interaction (FOCE-I) was used throughout the model-building procedure. Discrimination between models during the model building phase was based on standard visual diagnostics and the objective function value (OFV), calculated as proportional to twice the log-likelihood of the data. A reduction in OFV (∆OFV) of 3.84 and 6.64 was considered a significant improvement (p < 0.05 and p < 0.01, respectively) between two hierarchical models after inclusion of one additional parameter (one degree of freedom difference).

In the PK cohort, adherence to the previous 4 rounds of SMC during the season was not known. Therefore, we assumed complete adherence of the 4 rounds SMC prior to the PK cohort study, by including the previous doses when fitting the measured drug concentrations. This could potentially lead to a small under-estimation of the cut-off values in the adherence assessment (i.e. conservative approach).

Different methods were used to investigate the influence of data censoring below the lower limit of quantification (LLOQ). Omitting data measured below the LLOQ (M1-method), maximizing the likelihood to predict censored data to be below LLOQ (M3-method), and imputing the first concentration below LLOQ within a patient as half of the LLOQ (M6-method) were evaluated ^4^. The predictive performance of the different methods were assessed by comparing the proportion of predicted and observed data below the LLOQ, using categorical visual predictive checks ^4^.

Molar units of AQ and DEAQ concentrations were transformed into their natural logarithms. First-order elimination of AQ and DEAQ was assumed to take place from the central compartments. Parent and metabolite concentration-time data were fitted simultaneously, assuming 100% *in vivo* conversion of AQ to DEAQ. All possible combinations of structural distribution compartments were investigated, i.e. one-, two-, and three-compartment disposition models for both AQ and DEAQ. Inter-individual variability was added exponentially to all parameters (equation 1).

$\theta_{i}= \theta\cdot exp(\eta_{i,\theta})$ (1)

where, $\theta_{i}$ is the individual parameter estimate for the i^th^ individual, $\theta$ is the population estimate of the investigated parameter, and $\eta_{i,\theta}$ is the inter-individual variability (IIV) of the investigated parameter, assumed to be normally distributed with a zero mean and variance ω^2^. Relative bioavailability (F) was fixed to unity in the population, to allow investigation of the inter-individual variability of AQ absorption. The residual unexplained variability, assumed to be normally distributed with a zero mean and variance σ^2^, was modeled with an additive error on the log-transformed concentrations, which is approximately equivalent to an exponential residual error on an arithmetic scale.

**Covariate modelling**

Because the body weight of participants was not collected in the PK cohort study, we predicted the bodyweight using the data derived from a series of 3 community-based distributions between 2008 and 2010, and 6 community-based surveys between 2010 and 2016 in rural Niger, during the same season of the year as the PK study area [unpublished]. Anthropometric data (weight, height) and reported age were collected on a total of 127,256 children aged 6-60 months. The relationship between mean values of body weight with each age group in months were adequately (*R^2^* > 0.99) described by linear regression models for boys, girls and for the combined data of boys and girls (equation 2, 3 and 4). Based on these relationships, the predicted body weight of each child in both the PK cohort and case-control study were calculated from the reported age.

Boys,${BW}_{i}\left( kg \right)= 5.75+ 0.160\cdot Age \left( months \right)$ (2)

Girls: ${BW}_{i}\left( kg \right)= 5.13+ 0.162\cdot Age \left( months \right)$ (3)

Boys & girls: ${BW}_{i}\left( kg \right)= 5.46+ 0.162\cdot Age \left( months \right)$ (4)

Firstly, predicted body weight was evaluated in the model as a simultaneous incorporation of an allometric function on all clearance and distribution volume parameters, respectively (equation 5 and 6).

$\theta_{i}= \theta\cdot{(\frac{{BW}_{i}}{{BW}_{median}})}^{0.75}\cdot exp \left( \eta_{i,\theta} \right)$ (5)

$\theta_{i}= \theta\cdot(\frac{{BW}_{i}}{{BW}_{median}})\cdot\exp\left( \eta_{i,\theta} \right)$ (6)

where BW_i_ is the individually predicted body weight and BW_median_ is the predicted median body weight of the PK cohort population (i.e. 10 kg). Secondly, age-related enzyme maturation was evaluated on the clearance of AQ and DEAQ, respectively, using a saturation-type function (equation 7).

${CL}_{i}={CL}_{TV}\cdot\frac{Age}{{Age}_{50}+Age}\cdot{(\frac{{BW}_{i}}{{BW}_{median}})}^{0.75}\cdot\exp\left( \eta_{i,CL} \right)$ (7)

where ${CL}_{i}$ is the individually predicted clearance and ${CL}_{TV}$is the typical clearance value of the population. ${Age}_{50}$ is the age associated with reaching 50% of the clearance maturity.

Weight-for-age Z-score (WAZ) was calculated from the predicted average bodyweights and the WHO 2007 growth standard^5^ using the anthro package in R. The WAZ was investigated as a continuous covariate on all model parameters. Finally, gender was investigated on the all model parameters using a forward selection (p = 0.05) and backward elimination (p = 0.01) approach.

**Model evaluation**

Basic goodness-of-fit diagnostics were used to evaluate systematic errors and model misspecification. The sampling importance resampling (SIR) approach^6^ was used to calculate parameter uncertainty in the final population PK model (samples = 2,000, resamples = 1,000). The overall predictive performance of the final model was evaluated using simulation-based diagnostics (i.e. visual predictive checks, n = 2,000 simulations).

**Sensitivity and specificity of the adherence method**

Considering the simulated adherence as the gold standard, a 2-by-2 table was used to calculate sensitivity and specificity for different cut-off percentiles (5^th^ to 95^th^, intervals of 5%). For example, when using the 5^th^ percentile as a cut-off value, 95% of simulated fully adherent individuals had DEAQ concentrations above the cut-off value, resulting in a true negative value of 1,900 (95% × 2,000 simulations). The number of simulated non-adherent individuals with DEAQ concentrations below the cut-off value would be the true positive value (*eg.*, 4,200 out of 6,000 using the 5^th^ percentile cut-off). Sensitivity was defined as the proportion of patients with true non-adherence whom was categorized as non-adherent (true positive value/total number of simulated non-adherent individuals), and specificity was defined as the proportion of patients with true full adherence whom was categorized as adherent (true negative value/ total number of simulated adherent individuals).

**SUPPLEMENTARY FIGURES**


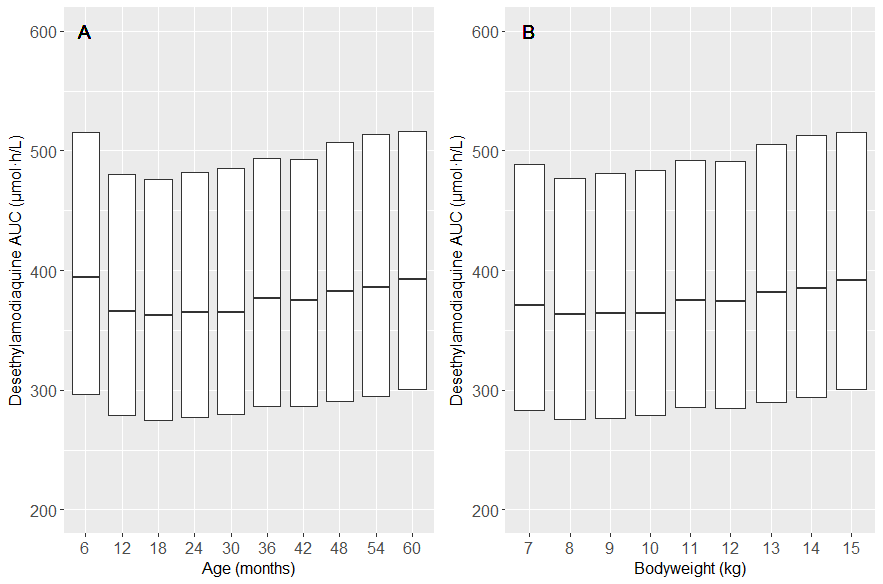


**Figure S1. The impact of age and bodyweight on the total exposure to desethylamodiaquine after a standard oral 3-day dosing of amodiaquine (10 mg/kg/day).**

A total of 5,000 children were simulated for each age (A), and bodyweight (B) group. Total exposure is presented as median values (25^th^ - 75^th^ percentiles). The derived relationship between age and bodyweight (Eq. 4) was used to assign typical bodyweights to children at different ages (A) and typical ages to children at different bodyweights (B)

**Figure S2. Goodness-of-fit of the final population pharmacokinetic model describing amodiaquine (A, B, C) and desethylamodiaquine (D, E, F).**

Observed plasma concentrations *vs.* individually predicted concentrations (A, D); conditionally weighted residuals *vs.* population predicted concentrations (B, E); conditionally weighted residuals *vs.* time (C, F). Solid red lines represent locally weighted least squares regressions.

**Figure S3. Flowchart of developed adherence methodology**

**DOT: directly observed therapy**

**REFERENCES**

1. Mwesigwa, J. *et al.* Pharmacokinetics of artemether-lumefantrine and artesunate-amodiaquine in children in Kampala, Uganda. *Antimicrob. Agents Chemother.* **54**, 52–59 (2010).

2. Hietala, S. F. *et al.* Population pharmacokinetics of amodiaquine and desethylamodiaquine in pediatric patients with uncomplicated falciparum malaria. *J. Pharmacokinet. Pharmacodyn.* **34**, 669–686 (2007).

3. Ali, A. M. *et al.* Population Pharmacokinetics of the Antimalarial Amodiaquine: a Pooled Analysis To Optimize Dosing. *Antimicrob. Agents Chemother.* **62**, (2018).

4. Bergstrand, M. & Karlsson, M. O. Handling data below the limit of quantification in mixed effect models. *AAPS J.* **11**, 371–380 (2009).

5. WHO Child growth standards. (2006). https://www.who.int/childgrowth/standards/weight_for_age/en/

6. Dosne, A.-G., Bergstrand, M., Harling, K. & Karlsson, M. O. Improving the estimation of parameter uncertainty distributions in nonlinear mixed effects models using sampling importance resampling. *J. Pharmacokinet. Pharmacodyn.* **43**, 583–596 (2016).

**NONMEM code for final population PK model**

$INPUT

ID ; Patient ID

TIME ; Time of sample

DV ; Dependent variable (natural logarithm of observed concentrations, nmol/L)

WT ; Body weight (kg, covariate)

AGE ; Age (month, covariates)

EVID ; Event ID record

MDV ; Missing dependent variable (1=missing)

AMT ; Dose amount (μmol)

CMT ; Compartment (1=dose, 2= amodiaquine, 3 = desethylamodiaquine)

$DATA

dataset.csv IGNORE=#

$SUBROUTINE

ADVAN5 TRANS1

$MODEL

COMP = (1) ; Dose

COMP = (2) ; Amodiaquine (AQ) central compartment

COMP = (3) ; Desethylamodiaquine (DEAQ) central compartment

COMP = (4) ; AQ peripheral compartment

COMP = (5) ; DEAQ peripheral compartment 1

COMP = (6) ; DEAQ peripheral compartment 2

$PK

;------------------------------------ Age covariate for AQ -------------------------------------------------------------------------

AGE50_AQ = THETA(13) ; Age to reach 50% of full maturation for AQ

MF_AQ = AGE / (AGE50_AQ + AGE) ; Age covariate relationship for AQ

;------------------------------------------------------------------------------------------------------------------------------------------

;------------------------------------ Age covariate for DEAQ ---------------------------------------------------------------------

AGE50_DEAQ = THETA(14) ; Age to reach 50% of full maturation for DEAQ

MF_DEAQ = AGE / (AGE50_DEAQ + AGE) ; Age covariate relationship for DEAQ

;------------------------------------------------------------------------------------------------------------------------------------------

TVCL = THETA(1) * ((WT/10)**0.75) * MF1 ; Population AQ clearance

CL = TVCL * EXP(ETA(1)) ; Individual AQ clearance

TVV2 = THETA(2) * (WT/10) ; Population AQ central volume

V2 = TVV2 * EXP(ETA(2)) ; Individual AQ central volume

TVKA = THETA(3) ; Population absorption rate constant

KA = TVKA * EXP(ETA(3)) ; Individual absorption rate constant

TVF1 = THETA(4) ; Population relative bioavailability

F1 = TVF1 * EXP(ETA4) ; Individual relative bioavailability

TVQ1 = THETA(5) * ((WT/10)**0.75) ; Population AQ inter-compartment clearance

Q1 = TVQ1 * EXP(ETA(3)) ; Individual AQ inter-compartment clearance

TVV4 = THETA(6) * (WT/10) ; Population AQ peripheral volume

V4 = TVV4 * EXP(ETA(6)) ; Individual AQ peripheral volume

TVCLM = THETA(7) * ((WT/10)**0.75)*MF2 ; Population DEAQ clearance

CLM = TVCLM* EXP(ETA(7)) ; Individual DEAQ clearance

TVV3 = THETA(8) * (WT/10) ; Population DEAQ central volume

V3 = TVV3 * EXP(ETA(8)) ; Individual DEAQ central volume

TVQ2 = THETA(9) * ((WT/10)**0.75) ; Population DEAQ inter-compartment clearance 1

Q2 = TVQ2 * EXP(ETA(9)) ; Individual DEAQ inter-compartment clearance 1

TVV5 = THETA(10) * (WT/10) ; Population DEAQ peripheral volume 1

V5 = TVV5 * EXP(ETA(10)) ; Individual DEAQ peripheral volume 1

TVQ3 = THETA(11) * ((WT/10)**0.75) ; Population DEAQ inter-compartment clearance 2

Q3 = TVQ3 * EXP(ETA(11)) ; Individual DEAQ inter-compartment clearance 2

TVV6 = THETA(12) * (WT/10) ; Population DEAQ peripheral volume 2

V6 = TVV6 * EXP(ETA(12)) ; Individual DEAQ peripheral volume 2

K12 = KA ; Absorption rate constant

K24 = Q1/V2 ; AQ distribution rate constant (COMP 2 --> 4)

K42 = Q1/V4 ; AQ distribution rate constant (COMP 4 --> 2)

K23 = CL/V2 ; AQ elimination rate constant (COMP 2 --> 3)

K35 = Q2/V3 ; DEAQ distribution rate constant (COMP 3 --> 5)

K53 = Q2/V5 ; DEAQ distribution rate constant (COMP 5 --> 3)

K36 = Q3/V3 ; DEAQ distribution rate constant (COMP 3 --> 6)

K63 = Q3/V6 ; DEAQ distribution rate constant (COMP 6 --> 3)

K30 = CLM/V3 ; DEAQ elimination rate constant (COMP 3 --> 0)

S2 = V2/1000 ; Scaling for AQ central volume

S3 = V3/1000 ; Scaling for DEAQ central volume

$ERROR

IF(F.GT.0) IPRED = LOG(F) ; Natural logarithm of predictions

IF (CMT.EQ.2) W = SQRT(SIGMA(1,1)) ; AQ residual error

IF (CMT.EQ.3) W = SQRT(SIGMA(2,2)) ; DEAQ residual error

IRES = IPRED – DV ; Individual residual error

IWRES = IRES / W ; Individual weighted residual error

IF (CMT.EQ.2) Y = IPRED + EPS(1) ; AQ additive residual error

IF (CMT.EQ.3) Y = IPRED + EPS(2) ; DEAQ additive residual error

$THETA ; Initial estimates of theta

(0, 101) ; 1. AQ clearance

(0, 314) ; 2. AQ central volume

(0, 2.85) ; 3. Absorption rate constant

(1 FIX) ; 4. Relative bioavailability

(0, 119) ; 5. AQ inter-compartment clearance

(0, 1820) ; 6. AQ peripheral volume

(0, 2.33) ; 7. DEAQ clearance

(0, 49.1) ; 8. DEAQ central volume

(0, 2.3) ; 9. DEAQ inter-compartment clearance 1

(0, 363) ; 10. DEAQ peripheral volume 1

(0, 4.34) ; 11. DEAQ inter-compartment clearance 2

(0, 98.1) ; 12. DEAQ peripheral volume 2

(0, 4.66) ; 13. Age to reach 50% of full maturation for AQ

(0, 2.42) ; 14. Age to reach 50% of full maturation for DEAQ

$OMEGA ; Initial estimates for omega

(0.0493) ; 1. AQ IIV clearance

(0.646) ; 2. AQ IIV central volume

(3.0) ; 3. IIV absorption rate constant

(0.141) ; 4. IIV relative bioavailability

(0 FIX) ; 5. AQ IIV inter-compartment clearance

(0 FIX) ; 6. AQ IIV peripheral volume

(0.0232) ; 7. DEAQ IIV clearance

(0 FIX) ; 8. DEAQ IIV central volume

(0 FIX) ; 9. DEAQ IIV inter-compartment clearance 1

(0.466) ; 10. DEAQ IIV peripheral volume 1

(0 FIX) ; 11. DEAQ IIV inter-compartment clearance 2

(0 FIX) ; 12. DEAQ IIV peripheral volume 2

$SIGMA ; Initial estimates of sigma

(0.688) ; 1. AQ residual variability

(0.0417) ; 2. DEAQ residual variability

$ESTIMATION POSTHOC MAXEVAL=9999 METHOD=1 INTER
