# Supplementary material for: Adherence and Population Pharmacokinetic Properties of Amodiaquine When Used for Seasonal Malaria Chemoprevention in African Children
Source: Clin Pharmacol Ther. 2019 Dec 31;107(5):1179–88. doi: 10.1002/cpt.1707 (PMC7232861; doi:10.1002/cpt.1707)
Supplement: Supplementary file 2 — Supplementary Material S1. EMERGE‐guidelines‐Checklist. [file CPT-107-1179-s002.docx]

**ESPACOMP Medication Adherence Reporting Guideline (EMERGE)^4,5^***Version 1 – 2018*

| **Section** | **Item No** | **Recommendation** | **Reported on  page No / line No** |
| --- | --- | --- | --- |
| **Minimum reporting criteria** |  |  |  |
|  | 1a | **Phases of medication adherence**: State the phase(s) of medication adherence studied (i.e. initiation, implementation, and persistence) and justify, where possible, the reasons the study focuses on this/these phase(s). | **Page 5/**  **line 80** |
|  | 1b | **Operational definition**: Provide the precise operational/working definition for each phase of medication adherence studied (i.e., initiation, implementation, and persistence). | **Page 5/**  **line 80** |
|  | 1c | **Measurement**: Specify the methods of measuring medication adherence (e.g., self-report, claims data, blood sampling, electronic monitoring). Consider each phase studied (i.e., initiation, implementation, and persistence), with details on the performance of the measures (e.g., validity, reliability, and potential bias). | **Page 15/**  **line 323** |
|  | 1d | **Results**: Describe the results of the analysis appropriate to each phase of medication adherence studied (i.e., initiation, implementation, and persistence). | **Page 8/**  **Line 163** |
|  |  |  |  |
| **Abstract** |  |  |  |
|  | 2a | Present in the abstract, in as much detail as space permits, information on the 4 minimum reporting criteria (i.e., items 1.a- 1.d). | **1.a Line 43**  **1.b Line 50**  **1.c Line 46**  **1.d Line 48** |

|  |  |  |  |
| --- | --- | --- | --- |
| **Background/introduction** |  |  |  |
|  | 3a | Summarize what is known about the topic with appropriate reference to the phase(s) of medication adherence (i.e., initiation, implementation, and persistence). | **Page 5/**  **Line 89** |
|  | 3b | Describe the rationale and/or framework guiding the medication adherence study (e.g., theoretical framework and implementation science model). | **Page 5/**  **Line 95** |
| **Study objectives or hypotheses** |  |  |  |
|  | 4a | State the study objectives or hypotheses with reference to the phase(s) of medication adherence studied and context (patient population and setting). | **Page 6/**  **Line 100** |
|  |  |  |  |
| **Methods** |  |  |  |
| **Design & participants** | 5a | Describe the setting in which the study was done. Refer to factors relevant to medication adherence, such as characteristics of the healthcare system, organization, and the team. | **Page 13/**  **Line 269** |
|  | 5b | State whether medication adherence was an eligibility criterion (e.g., inclusion/exclusion). If so, define the measures and rules used. | **Not applicable** |
|  | 5c | Describe routine care related to the management of medication adherence, if applicable (e.g. routine assessment of medication adherence, adherence support programs, and provider training). | **Not applicable** |
| **Measurement** | *Please refer to item 1.c. in addition to the “Measurement” item below* | |  |
|  | 6a | Measurement methods can themselves affect medication adherence (e.g., questionnaires, blood sampling, and electronic monitoring). Address this problem as appropriate. | **Page 15/**  **Line 323** |
| **Intervention (where applicable)** | 7a | For intervention and comparator groups, describe each relevant level of the medication adherence intervention (e.g., healthcare system, organization, and provider and patient/caregiver). | **Not applicable** |
|  | 7b | Describe any implementation strategy that contributes to the translation (e.g., uptake, delivery, and sustainability) of the medication adherence intervention in clinical practice, if applicable. | **Not applicable** |
| **Statistical analysis** | 8a | If medication adherence is an outcome variable, justify the statistical methods, given the characteristics of the variable (e.g., phases of medication adherence, data type, statistical distribution, data censoring, longitudinal dependence). | **Page 15, 17/**  **line 323, 351** |
|  | 8b | If medication adherence is an explanatory variable, describe how it is related to the outcome(s) (e.g., causal pathway, temporal sequence). | **Not applicable** |
|  |  |  |  |
| **Results** |  |  |  |
|  | *Please refer to item 1.d in addition to the “Results” items below* | |  |
|  | 9a | Determine whether non-participation and/or dropout are associated with non-adherence, and provide any relevant data. | **Not applicable** |
|  | 9b | Present sample characteristics relevant to medication adherence (e.g., those related to socio-demographics and therapy, condition, patient, caregiver, healthcare team/healthcare system). | **Page 13/**  **Line 259** |
|  |  |  |  |
| **Discussion** |  |  |  |
|  | 10a | Discuss study strengths and limitations with reference to the phase(s) of medication adherence, where applicable (i.e., initiation, implementation, and persistence). | **Page 12/**  **Line 247** |
|  | 10b | Discuss the study findings in the context of existing evidence on medication adherence (e.g., theory, measurement, intervention effects). | **Page 11/**  **Line 217** |
|  | 10c | Discuss the generalizability (external validity) of the study findings with reference to the phase(s) of medication adherence, where applicable (i.e., initiation, implementation, and persistence). | **Page12/**  **line 239** |
